# Management of persistent anal canal carcinoma after combined-modality therapy: a clinical review

**DOI:** 10.1186/1748-717X-9-39

**Published:** 2014-01-28

**Authors:** Daniela Musio, Francesca De Felice, Nicola Raffetto, Vincenzo Tombolini

**Affiliations:** 1 Department of radiological, oncological and anatomo-pathological science, “Sapienza” University, Viale Regina Elena, Rome, Italy

## Abstract

Anal canal carcinoma is a rare gastro-intestinal cancer. Radiochemotherapy is the recommended primary treatment for patients with non-metastatic carcinoma; surgery is generally reserved for persistent or recurrent disease. Follow-up and surveillance after primary treatment is paramount to classify patients in those with complete remission, persistent or progressive disease. Locally persistent disease represents a clinically significant problem and its management remains subject of some controversy.

The aim of this systematic review is to summarise recommendations for the primary treatment of anal canal carcinoma, to focus on the optimal time to consider residual disease as genuine persistence to proceed with salvage treatment, and to discern how this analysis might inform future clinical trials in management in this class of patients.

## Introduction

Carcinoma of the anal canal is a human papilloma virus associated cancer affecting both men and women. It accounts for only 2% of gastro-intestinal malignancies, but its incidence has been increasing for the past 30 years, with, in 2013, estimated worldwide new cancer cases of 7.060 in both sexes [1]. Despite the rarity of anal canal cancer, it represents a successful model for the multimodality treatment in radiation oncology research.

Until the 1980s, surgery was the treatment of choice; nowadays it is reserved for patients with persistent or recurrent disease. Due to superior local control and survival, and due to a better quality of life, prospective randomized trials have established that the submission of a combination of radiotherapy (RT) and chemotherapy is the international standard of care for patients with anal canal cancer [2-4]. Accurate tumour evaluation after combined-modality therapy is thus essential for prognostic information and for patient’s care. Questions remain about the most effective management planning after combined-modality therapy. Based on the results of the ACT-II study [5], the NCCN guidelines recommended a clinical observation – digital rectal exploration, inguinal node palpation and anoscopy – as long as there is no evidence of progressive disease [6]. Whereas, the European Society for Medical Oncology guidelines suggests that MRI can be used to complement clinical assessment in response evaluation [7]. In light of the difficulties in obtaining clear recommendations, this review discusses conceptual issues pertinent to anal carcinoma treatment, with specific details in the management after primary combined therapy.

### Primary treatment of anal canal carcinoma

Since the pioneering work by Nigro et al. [8] in the 1974, the primary therapy of anal canal carcinoma has shifted from radical surgery to combined radiochemotherapy (CRT), because of the chance of cure with a best quality of life. Local excision could be considered for small well-differentiated carcinomas of the anal margin without evidence of sphincter involvement [7]. Randomized phase III trials have demonstrated the benefit of CRT over RT alone, with a five-year disease free survival in the range of 67–73% [2-4]. Radiation therapy plus concurrent 5-fluorouracil (5-FU) and mitomycin C (MMC) represents the preferred standard of care, due to the clinically and statistically significant impact on survival, both overall and disease free [5,9]. No randomized controlled trials have found a chemotherapy regimen substantially more effective at improving local and distant control. Two randomized trials tested whether cisplatin (CDDP) could be used instead of MMC, but results failed in their objective [5,10]. The US Gastrointestinal Intergroup RTOG 98–11 trial data [10] did not show a statistically significant difference in disease-free survival between 320 patients who received RT with 5-FU plus CDDP versus 324 patients submitted to 5-FU plus MMC (60% vs 54%, p-value 0.17). Both colostomy-free survival and overall survival were improved by CRT with MMC/5-FU as compared to CRT with CDDP/5-FU (72% vs 65% and 78% vs 71%, respectively). The ACT II [5] enrolled 940 patients randomly assigned to RT and concomitant MMC/5-FU or CDDP/5-FU. Results reported no difference on progression-free survival between the two chemotherapy strategies investigated (HR 0.95, 95% CI 0.75–1.19; p = 0.63), such as on overall survival and colostomy-free survival (HR 1.05, 95% CI 0.80-1.38; p = 0.70; and 73% vs 75%, respectively).

Nowadays, RT with 5-FU and MMC remains the standard practice in anal canal carcinoma, and, however it is a rare situation, CDDP could be used in those patients in which MMC is contraindicated.

### Tumour regression

Tumour regression in defined as the total disappearance of tumour with a normal anus mucosa [11]. The median time to complete clinical regression after CRT is about 12 weeks (range 2 – 36 weeks) [12,13]. But tumour regression may be slower: some cancer could take up to 48 weeks to disappear [12], other to 72 weeks [14]. Although the rapidity of the clinical response to therapy represents a prognostic factor [11,15], these data confirmed that a correct management after combined-modality therapy is paramount.

Following primary treatment patients are re-evaluated. Current guidelines include serial digital rectal exploration, inguinal node palpation and anoscopy, with biopsy of clinically evidence of progressive disease after CRT [6]. Lesions are classified according to their response in complete regression, persistent disease or progressive disease.

Clinical and imaging assessment of the radiation treated field is often complicated by acute toxicity, such as mucositis, that can persist for several weeks. In addition, the clinical and imaging appearance of radiation reactions can mimic persistent or recurrent disease. The obvious limitation of clinical evaluation is the subjectivity of the examination and the absence of treatment response information for any deep and/or non-palpable disease. But routine biopsy should not be performed, because irradiated mucosa does not have the same ability to heal as normal mucosa and persisting discomfort, like bleeding or proctitis, could be observed [5]. Moreover, pathologic interpretation of post-treatment biopsy can be difficult due to significant treatment-related alterations [16].

Therefore the timing of the identification of treatment failure remains controversial. Patients who had not achieved a rapid complete response to treatment could benefit from a longer follow-up for tumour regression and a salvage surgery can thereby be avoided. Currently an arbitrary cut-off of 6 months after CRT is considered adequate to accuracy of the biopsy and, consequently, to distinguish patients with persistent disease from those with complete response [17]. If no regression of disease is observed by 6 months a salvage abdominal-perineal resection should be considered [6]. But in several cases, the 6-month window could be too early to allow for a complete tumour response to occur [12,14]. Results from the UKCCCR randomised trial showed that 77% of patients, who had not complete tumour regression at the 6-week assessment, achieved a complete response of primary tumour to CRT treatment with longer follow-up [18]. Persistent disease may continue to regress even at 26 weeks after the end of CRT. In these cases, it is possible that patients are submitted to abdominal-perineal resection but no cancer is found on histopathological examination.

Previous literature has attempted to elucidate the better treatment that may assure better rates of local control and survival of patients with anal canal carcinoma. Randomised phase III trials’ primary end-point was to demonstrate the benefit of CRT over radiotherapy, evaluating time to first loco-regional relapse (see Table 1). Most studies have attempted to define factors predictive of outcome variables, such as age, tumour stage and response to therapy [11,15]. Thus timing of tumour response was never clearly defined. Randomised data reported the presence of residual tumour evaluated within 6 weeks following the end of treatment. It was considered as initial local failure and a salvage surgery was proposed, but specific histological data are not available and it is difficult to argue if an early intervention was performed.

**Table 1:** End-points of randomized trials

**Trial**	**Patients**	**Study design**	**Primary end-point**	**Secondary end-point**
ACT I [2]	560	CRT vs RT	LC; RFS; CFS; OS	-
Intergroup [3]	310	CRT vs RT	LC; CFS; OS; DFS	-
EORTC [4]	110	CRT vs RT	LC	OS; CFS
UKCCCR [18]	856	CRT vs RT	LC	OS; morbidity

*LC* local control; RFS: relapse free survival, *CFS* colostomy free survival, *OS* overall survival, *DFS* disease free survival.

Single institute retrospective analysis reported their experience with salvage surgery after failed CRT therapy, but time-details of biopsy or of clinical determination of residual/persistent disease are not available [19-21].

The optimal time for surgical intervention remains uncertain; avoidance of unnecessary overtreatment and excessive delay in treatment are both important, and an observation period of 6 months is a reasonable balance commonly used.

### Response evaluation

Response evaluation after CRT represents a significant problem and which is the optimal recommendation is still debate. Traditionally response to CRT is assessed clinically, because treatment related changes can cause difficulties in response interpretation. As both endoanal ultrasound and magnetic resonance imaging (MRI) play an established role in the accurate locoregional staging of primary lesion, their role in post-treatment evaluation is more debatable [22]. Response as defined by Response Evaluation Criteria in Solid Tumours (RECIST criteria) at 6–8 weeks is a commonly employed end-point in phase II and phase III trials, but it is useful only to achieve a predictive evaluation. Oedema or scar tissue is difficult to distinguish from persistent disease on endoanal ultrasound. Response evaluation of treated disease with MRI can lead to false persistent disease due to RT-related tumour fibrosis. Several studies were proposed to establish the real prognostic time to evaluate treatment response, but all are based on small cohort of patients. Giovannini et al. [23] analyzed the follow-up of 147 patients with anal canal disease after CRT. They reported that 16–20 weeks after the end of therapy represent a sufficient time to classify treatment response by endoanal ultrasound, due to resolution of oedema. In Tarantino et al. study [24] 12 patients, with a biopsy-proven squamous-cell anal carcinoma, were submitted to surgery or CRT after endoanal ultrasound evaluation. In surgery group, pathological staging was correlated with ultrasound clinical staging; while for CRT patients, endoanal ultrasound was compared to local biopsies performed two - four months after the end of treatment. Ultrasound evaluations correlated with surgical and biopsy findings. Authors concluded that endoanal ultrasound could be used to accurately determine the response of the squamous-cell anal carcinoma to multimodality therapy.

The high contrast resolution of pelvic MRI makes it an ideal modality for response assessment, understanding the pattern of tumour regression and monitoring treatment response. However few data on optimal time to perform MRI and to evaluate treatment response are available [13]. Certainly MRI has a specific role in the evaluation of recurrent disease following RT, considering its specific signal characteristic – high signal intensity relative to skeletal muscle on T2-weighted images, and low to intermediate signal intensity on T1-weighted images – [25]. A stabilization of signal intensity abnormality one year after the end of CRT may indicate a treatment success. However this observation needs to be verified in clinical studies with a great number of patients [26].

Metabolic and functional imaging techniques, such as diffusion-weighted MRI (DW-MRI) or positron emission tomography with F-18 fluorodeoxyglucose (FDG-PET), should be used to assess therapeutic response, but further studies are awaited to state stronger information.

DW-MRI may directly measure the changes in tumour aggressiveness, evaluating tumour angiogenesis and cellularity. The treatment response using DW parameter – apparent diffusion coefficient (ADC) value – has been shown to be predictive of early therapeutic response in a large variety of tumours, including head and neck tumours, pancreatic tumours, cervical tumours, and rectal cancer, but its potentiality as imaging biomarker in anal canal carcinoma is not still tested [27,28]. Pre- and post-treatment ADC value should be considered to address the use of DW-MRI as an alternative approach to the management of persistent disease.

FDG-PET may potentially provide further information of tumour glucose uptake, before and after cancer therapy. Its utility in the pre-treatment evaluation of anal canal carcinoma is well documented [29,30] and the NCCN treatment guidelines now consider FDG-PET an optional diagnostic exam for staging primary disease [6]. Schwarz et al. [16] evaluated the metabolic response to therapy using FDG-PET, in 53 consecutive patients with anal canal. Post-treatment FDG-PET was performed 0.9-5.4 months (mean, 2.1; median, 2.0) after treatment: persistent FDG uptake was confirmed as residual disease by biopsy in 67% of patients. Additional post treatment studies are necessary to determine the optimal timing of post FDG-PET evaluation, to reduce false-positive results.

MRI, with or without DW, and FDG-PET should be performed if there is a clinical suspicion of disease persistence, but advances in imaging are necessary to improve medical decision making in the management of anal cancer.

### Future perspectives

The immediate future of research in anal canal carcinoma will be two-fold. One will be of diagnostic impact, determining what combination of metabolic assays (ADC, FDG-PET) is the most robust, sensitive and specific for residual disease after treatment. The second is determining the biochemical and biological effects of molecular inhibitors agents in tumour cells, in order to validate targeted therapies for this carcinoma. There is an opportunity for study of newer prognostic factor, such as tumour-infiltrating lymphocytes, in attempt to improve immunotherapeutic strategies [31]. No ongoing trials explore the optimal timing to evaluate treatment response in anal canal carcinoma.

The right identification of post-treatment response is paramount to patient’s management and it should be considered an hypotheses to test in a prospective trial.

A cut-off > 6 months seems to have a potential value, in patients with a partial response to treatment. We have evaluated the effect of CRT in 15 patients, with histologically proven anal squamous cell carcinoma, clinically staged on anoscopy and whole body CT as T1 (2 patients), T2 (9 patients), T3 (3 patients), T4 (1 patient); 5 patients had positive lymph nodes. Response to CRT was assessed clinically. In case of an uncertain evaluation, patients underwent anoscopy and/or abdominal-pelvic MRI. Six months after the end of treatment, all patients had digiatal examination, anoscopy and whole body CT, as at baseline. Discordance in response evaluation was noticed in 3 patients: negative clinical examination and positive imaging results were found in 2 patients, positive digital examination and complete imaging response in 1 patient. Abdominal-perineal resection was done and no tumour in the operative specimen was found. These facts suggested us the need for longer timing before surgical approach, to potentiate the beneficial effect of CRT and to reduce imaging treatment artificial. We have begun to use a cut-off of 8 months, in patients who had not achieved a rapid complete response to CRT. Our experience is too restricted to be considered a statement of evidence. Further investigation in a larger number of patients is necessary.

## Conclusions

A multidisciplinary approach is necessary in patients with anal canal carcinoma. The curative potential role of CRT is well documented. Follow-up clinical evaluations are recommended, but decision to base the management of persistent anal canal carcinoma after combined-modality therapy is not clearly progressing. Recent findings indicate that a complete clinical response occurs in the majority of patients, within 6-month the end of CRT. Patients with evidence of persistent disease without proven loco-regional progression should benefit from a close follow-up to evaluate if tumour regression occurs.

## Competing interests

The authors declare that they have no competing interests.

## Authors’ contributions

DM defined the study addressed by the manuscript. DM and FDF carried out the data, analyzed the data, defined the conclusions and wrote the paper. DM, FDF and VT discussed analyses and final results. DM, FDF, VT and NF revised the manuscript. All authors read and approved the final manuscript.
